# Effects of the Amylose/Amylopectin Ratio of Starch on Borax-Crosslinked Hydrogels

**DOI:** 10.3390/polym16162237

**Published:** 2024-08-06

**Authors:** Kai Lu, Rudy Folkersma, Vincent S. D. Voet, Katja Loos

**Affiliations:** 1 Macromolecular Chemistry and New Polymeric Materials, Zernike Institute for Advanced Materials, University of Groningen, Nijenborgh 3, 9747 AG Groningen, The Netherlands; k.lu@rug.nl; 2 Circular Plastics, Academy Technology & Innovation, NHL Stenden University of Applied Sciences, Van Schaikweg 94, 7811 KL Emmen, The Netherlands; rudy.folkersma@nhlstenden.com

**Keywords:** starch, amylose/amylopectin, hydrogel, borax, dynamic borate ester bonds

## Abstract

Herein, we simultaneously prepared borax-crosslinked starch-based hydrogels with enhanced mechanical properties and self-healing ability via a simple one-pot method. The focus of this work is to study the effects of the amylose/amylopectin ratio of starch on the grafting reactions and the performance of the resulting borax-crosslinked hydrogels. An increase in the amylose/ amylopectin ratio increased the gel fraction and grafting ratio but decreased the swelling ratio and pore diameter. Compared with hydrogels prepared from low-amylose starches, hydrogels prepared from high-amylose starches showed pronouncedly increased network strength, and the maximum storage modulus increased by 8.54 times because unbranched amylose offered more hydroxyl groups to form dynamic borate ester bonds with borate ions and intermolecular hydrogen bonds, leading to an enhanced crosslink density. In addition, all the hydrogels exhibited a uniformly interconnected network structure. Furthermore, owing to the dynamic borate ester bonds and hydrogen bonds, the hydrogel exhibited excellent recovery behavior under continuous step strain, and it also showed thermal responsiveness.

## 1. Introduction

Hydrogels consist of a three-dimensional structure made of physically or chemically crosslinked hydrophilic polymers that can absorb and retain plenty of water in the substitutional spaces between chains without disintegrating [1,2]. Owing to these unique characteristics, these materials have been widely used in various fields, such as hygiene, agriculture, food additives, drug delivery, and wastewater treatment [3,4,5,6,7]. However, most conventional hydrogels are derived from petroleum-based polymers with non-biodegradability, high cost, and potential toxicity, and their widespread use may cause environmental pollution [8,9]. Therefore, various natural polysaccharides have been used for the development of hydrogels because they are cost-effective, can be biodegradable, and are nontoxic [10,11].

Among these natural polysaccharides, starches have attracted increasing research interest because they are abundant, renewable, biodegradable, and inexpensive [12,13]. Starch molecules contain a large number of hydroxyl groups and can be easily crosslinked to form hydrogels [14,15]. Moreover, starches consist of two main glycosidic macromolecules: amylose and amylopectin [16,17]. Amylose is a linear polymer that mainly consists of α-1,4-linked glucose units with molecular weights ranging from 1.3 × 10^5^ to 5 × 10^5^ g/mol. Amylopectin is a branched polymer that consists of short α-1,4 chains branched via α-1,6 linkages. The molecular weight of amylopectin is approximately 10^7^ to 10^9^ g/mol [18,19,20,21]. The ratio of amylose to amylopectin can significantly influence the physicochemical properties of starches, such as the solubility, viscosity, and retrogradation, and therefore affect the properties of starch-based hydrogels [22,23]. Bao et al. studied the effect of the amylose/amylopectin ratio on the rheological behaviors of starch-based hydrogels, and the results showed that the storage modulus (*G*′) of hydrogels increased with increasing amylose content [24]. Zhang et al. prepared superabsorbent polymers by the copolymerization of monomers onto amylose and amylopectin and reported that the water absorption capacity and grafting ratio of amylose graft copolymers are superior to those of amylopectin graft copolymers [25].

Starch-based hydrogels usually suffer from poor mechanical properties, and they easily deform and cannot recover when subjected to external damage, which limits their widespread application [26,27]. Therefore, developing starch-based hydrogels with simultaneous remarkable mechanical properties and self-healing properties is highly important for extending their lifetime. At present, self-healing hydrogels are mainly prepared by introducing dynamic covalent and/or noncovalent bonds [28]. The dynamic covalent bonds mainly include imine bonds, disulfide bonds, hydrazone bonds, and borate ester bonds [29,30,31,32]. Noncovalent bonds mainly consist of π-π stacking, host–guest interactions, electrostatic interactions, and hydrogen bonds [33,34,35,36]. Borax is an efficient and reversible crosslinker, and it dissociates into equal quantities of trigonal planar boric acid B(OH)_3_ and tetrahedral borate ions (B(OH)_4_^−^) in water. The concentration of B(OH)_4_^−^ was almost twice the initial borax concentration [37,38]. The resulting borate ions can form dynamic borate ester bonds with the hydroxyl groups of starch, and the dynamic reversible nature of these bonds endows the hydrogels with excellent self-healing properties and stimuli responsiveness by influencing the crosslink density of hydrogels [39,40,41]. Lu et al. prepared a hydrogel with multiresponsive and self-healing properties, which were due to the formation of interchain dynamic didiol-borax complexations between the B(OH)_4_^−^ and the OH groups on the sides of the PVA/MFC [32]. Spoljaric et al. prepared hydrogels by blending nanofibrillated cellulose, PVA, and borax. The results showed that the addition of borax improved the mechanical performance of the hydrogels [42]. Therefore, we hypothesized that borax can be used to develop self-healing and mechanically strong starch-based hydrogels.

In this work, we have developed a simple one-pot method to prepare borax-crosslinked starch-based hydrogels with self-healing and thermal responsiveness. Corn starches with different amylose/amylopectin ratios (waxy, 4.3/95.7; maize, 27/73; Gelose 50, 50/50; and Gelose 80, 80/20) were used to study their effect on the grafting reactions as well as the performance of the resulting starch-based hydrogels. The swelling ratio; gel fraction; and rheological, microstructural, self-healing, thermosensitive, and thermal properties of the prepared hydrogels were systematically studied. Overall, we believe that the present research could aid in understanding the effect of the starch molecular structure on the crosslinking behavior of borax with starch, which in turn influences the mechanical and self-healing properties of starch-based hydrogels and facilitates the development of sustainable hydrogels.

## 2. Materials and Methods

### 2.1. Materials

Corn starches with different amylose/amylopectin ratios were used. Waxy (4.3% amylose content) was obtained from Fuyang Co., Ltd. (Dezhou, China). Maize (27% amylose content) was purchased from Sigma Aldrich (Darmstadt, Germany). Gelose 50 and Gelose 80 (with 50 and 80% amylose contents, respectively) were obtained from Penford (Sydney, Australia). Acrylamide (AM, CH_2_=CHCONH_2_), borax (Na_2_B_4_O_7_·10H_2_O), ceric ammonium nitrate (CAN, (NH_4_)_2_Ce(NO_3_)_6_), and ethanol were purchased from Sigma Aldrich (Darmstadt, Germany). Distilled water was used in the preparation of all the solutions. All the chemicals were of analytical grade and used without further purification.

### 2.2. Sample Preparation

**Scheme 1:**
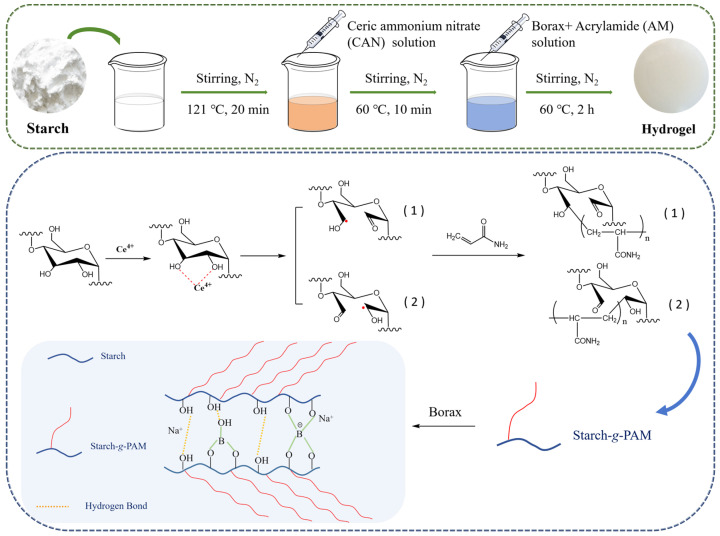
Method and mechanism of synthesis of borax-crosslinked hydrogels.

The borax-crosslinked hydrogels from different starches were prepared as shown in Scheme 1. A total of 5.00 g of corn starch was dissolved in 80 mL of distilled water under stirring for 20 min at 121 °C under a nitrogen atmosphere to obtain a fully gelatinized starch solution. Subsequently, 5 mL of freshly prepared CAN solution containing 0.70 g of CAN was added to facilitate free radical formation on starch at 60 °C for 10 min under a nitrogen atmosphere. Afterwards, a pre-mixed solution containing 15.00 g of acrylamide (AM), 0.50 g of borax, and 49.50 mL of distilled water was added at 60 °C to react for 2 h under a nitrogen atmosphere. Finally, the resultant product was washed with distilled water and then soaked in acetone to remove ungrafted monomers and unreacted borax. The hydrogels prepared from different starches were named Waxy-G, Maize-G, G50-G, and G80-G. For the control test, a hydrogel prepared from G80 without borax was named G80-CG. All the samples with various compositions are listed in Table 1.

### 2.3. Characterization Methods

#### 2.3.1. Fourier Transform Infrared Spectroscopy (FTIR)

An FTIR analysis of native starches and hydrogels was performed using a vertex 70 Bruker spectrometer in attenuated total reflectance (ATR) mode. The spectra were recorded at a resolution of 4 cm^−1^ in the range of 4000 to 400 cm^−1^ with 64 scans.

#### 2.3.2. H-NMR Spectroscopy

Proton nuclear magnetic resonance (^1^H-NMR; 600 MHz) measurements were recorded on a Bruker Ascend NMR600 spectrometer. The deuterated dimethyl sulfoxide (DMSO-*d*_6_, 99.9%) and deuterium oxide (D_2_O, 99.9%) were purchased from Sigma-Aldrich (Darmstadt, Germany). The native starches were dissolved in DMSO-*d*_6_, while the hydrogels were dissolved in D_2_O. To completely dissolve the sample, the samples were kept in an oven (65 °C) for 2 h before the measurement.

#### 2.3.3. X-ray Diffraction (XRD)

The X-ray patterns of the native starches and hydrogels were obtained using a Bruker D8 Advanced apparatus. The diffractograms were obtained at a scanning range of 5–50° (2θ) at 40 kV and 40 mA using Cu Kα radiation (λ = 0.1542 nm).

#### 2.3.4. Scanning Electron Microscopy (SEM)

The prepared hydrogels were freeze-dried and then coated with gold to make the samples conductive. The morphological structure of the samples was determined on a Philips XL30 field emission gun scanning electron microscope with EDAX EDS/EBSD detectors operating at an accelerating voltage of 10 kV. The images were analyzed with Nanomeasure software to determine the pore size distribution of the hydrogels.

#### 2.3.5. Gel Fraction

The gel fraction was measured by the following procedure outlined by Rana [43]. The synthesized hydrogels were dried at 60 °C, weighed (*W_i_*), and then soaked in distilled water for 3 days to a constant weight to remove the soluble parts. The hydrogels were dried again at 60 °C and weighed (*W_d_*). The gel fraction (*GF*) was calculated by

(1) $$GF(\%)=\frac{W_{d}}{W_{i}}\times 100$$

where *W_i_* is the initial dry weight of the hydrogel and *W_d_* is the final drying weight after dipping in water. All the experiments were performed in triplicate, and the average values are reported.

#### 2.3.6. Swelling Ratio

The swelling ratio of the hydrogels was measured by immersing the as-weighed (*W_i_*) samples in distilled water at room temperature until swelling equilibrium was reached [44]. Then, the swollen hydrogel was removed and weighed (*W_s_*), and the swelling ratio (*SR*) of the hydrogels was calculated by

(2) $$SR=\frac{W_{s}-W_{i}}{W_{i}}$$

where *W_i_* is the initial dry weight of the hydrogel and *W_s_* is the weight of the hydrogel in a swollen state. All the experiments were performed in triplicate, and the average values are reported.

#### 2.3.7. Thermogravimetric Analysis (TGA)

A thermal analysis of the native starches and hydrogels was evaluated using a TA instrument (5500) under a nitrogen atmosphere (N_2_) in the range of 30–700 °C at a heating rate of 10 °C/min.

#### 2.3.8. Rheological Properties

The rheological properties of the hydrogels were analyzed with an Anton Paar MCR302 Rheometer (Anton Paar, Ashland, VA, USA) with a parallel plate geometry of 25 mm in diameter. The dynamic strain sweep experiments were carried out with the strain amplitude γ varying from 0.1 to 100% at an angular frequency of ω = 10 rad/s. The storage modulus (*G*′) was recorded to define the linear viscoelastic region in which the storage modulus is independent of the strain amplitude (γ). For the dynamic frequency sweep experiments, the storage modulus (*G*′) and loss modulus (*G*″) of the hydrogels were recorded with an angular frequency ω ranging from 0.1 to 100 rad/s at a strain of γ = 1% in the linear viscoelastic region.

#### 2.3.9. Self-Healing Property

To investigate the self-healing property of the hydrogel, the hydrogel was cut into two parts and brought into contact at room temperature. The *G*′ and *G*″ for the original and self-healing G80-G hydrogels were measured through a dynamic time sweep test at ω = 10 rad/s and γ = 1% within 0 to 600 s. The recovery property of the G80-G hydrogel was evaluated using a continuous step strain test, which was performed as follows: γ = 1% (700 s) → γ = 100% (700 s) → γ = 1% (700 s) → γ = 100% (700 s) → 1% (900 s) at ω = 10 rad/s.

#### 2.3.10. Thermosensitivity Property

To investigate the thermosensitivity of the G80-G hydrogel, a temperature sweep was measured through a heating–cooling–heating process (20–95–20–95 °C) at ω = 10 rad/s and γ = 1%, where the heating and cooling rates were 5 °C/min.

#### 2.3.11. Statistical Analyses

The means and standard deviations were calculated for the gel fraction, swelling ratio, and SEM measurements. The statistical analyses were performed using one-way analysis of variance (ANOVA) with Duncan’s multiple range test in SPSS software (version 26, IBM, New York, NY, USA). The results with *p* < 0.05 were considered significantly different.

## 3. Results and Discussion

### 3.1. Hydrogel Morphology

**Figure 1:**
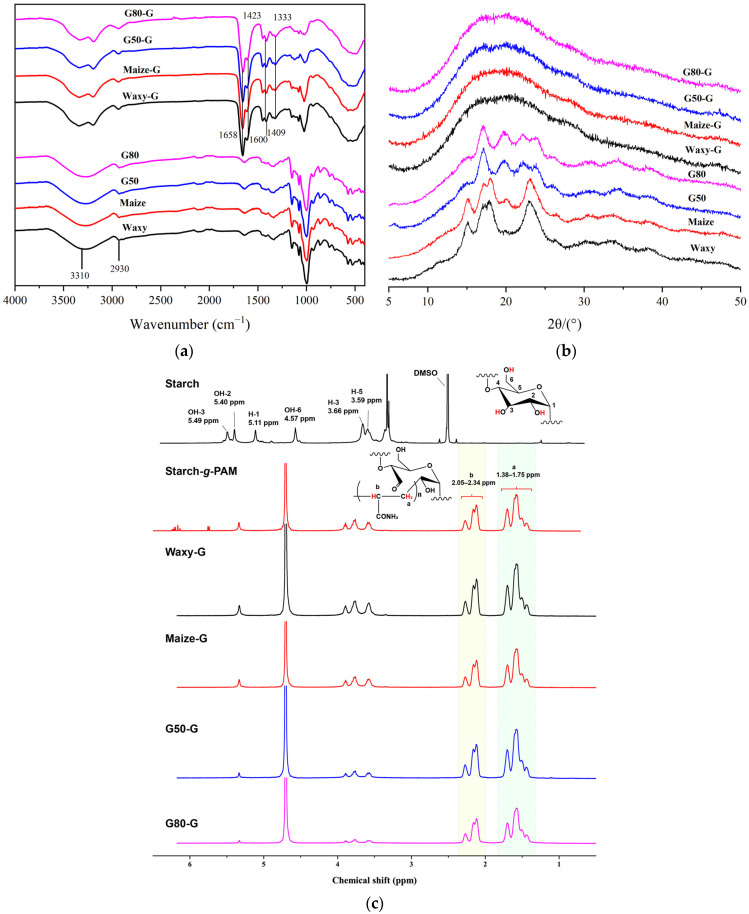
(**a**) FTIR spectra of starch and crosslinked hydrogels (Waxy-G, Maize-G, G50-G, and G80-G); (**b**) XRD patterns of native starches and crosslinked hydrogels (Waxy-G, Maize-G, G50-G, and G80-G); and (**c**) ^1^H-NMR spectra of starch, starch-*g*-PAM, and crosslinked hydrogels (Waxy-G, Maize-G, G50-G, and G80-G).

Starch-based hydrogels with varying amylose/amylopectin ratios were successfully prepared by using borax as a crosslinker. The FTIR spectra of the native starches and the Waxy-G, Maize-G, G50-G, and G80-G hydrogels are shown in Figure 1a. For the native starches, the characteristic absorption bands at 3310 cm^−1^ and 2930 cm^−1^ corresponded to the O-H stretching vibrations and C-H stretching vibrations, respectively. The peaks at 1625 cm^−1^, 1152 cm^−1^, and 1148 cm^−1^ could be attributed to the H-O bending vibrations, C-O-C stretching vibrations, and glycosidic bond vibrations, respectively. In addition, the peak at 1643 cm^−1^ corresponded to the O-H bending vibration of the water molecules in the starch [45,46]. The spectra of the prepared hydrogels displayed new characteristic peaks at 1658 cm^−1^, 1600 cm^−1^, and 1409 cm^−1^, which arose from the C=O stretching, N–H bending, and C–N stretching, respectively. These characteristics are characteristic of the -CONH_2_ group contained in acrylamide [47]. Moreover, the peaks at 1423 cm^−1^ and 1333 cm^−1^ were attributed to the asymmetric stretching relaxation of the B-O-C bonds, indicating the formation of borate ester bonds between the borax and the hydroxyl groups of the starch backbone, which led to a reduced intensity of the broad peak at 3310 cm^−1^ [44,48]. The XRD patterns of the native starches and the Waxy-G, Maize-G, G50-G, and G80-G hydrogels are presented in Figure 1b. Waxy and maize exhibited peaks at 15°, 17°, 18°, and 23°, which revealed the formation of an A-type crystallinity pattern. However, G50 and G80 exhibited typical B-type crystallinity patterns, with peaks at 5.6°, 17°, 22°, and 24° [49,50]. After the reaction, the XRD patterns of the starch hydrogels changed to amorphous structures because the typical peaks of native starch crystallites disappeared, with only a blunt amorphous peak at 20°. This phenomenon indicated that the strong interaction between starch chains and borate ions could suppress the retrogradation of amylose and/or amylopectin in starch, leading to a decrease in the number of starch crystallites.

Figure 1c shows the ^1^H-NMR spectrum of the native starches, starch-*g*-PAM, and the Waxy-G, Maize-G, G50-G, and G80-G hydrogels. The chemical shifts at 5.40, 5.49, and 4.57 ppm could be attributed to the starch protons of OH-2, OH-3, and OH-6, respectively, while those at 5.11, 3.66, and 3.59 ppm could be assigned to the H-1, H-3, and H-5 signals from the starch [51]. After the reaction, the ^1^H-NMR spectrum of the starch hydrogels showed signals at 1.38–1.75 ppm and 2.05–2.34 ppm, corresponding to the protons -CH_2_ and -CH in the PAM structure [52]. These results confirmed the effective grafting of acrylamide onto the starches.

**Figure 2:**
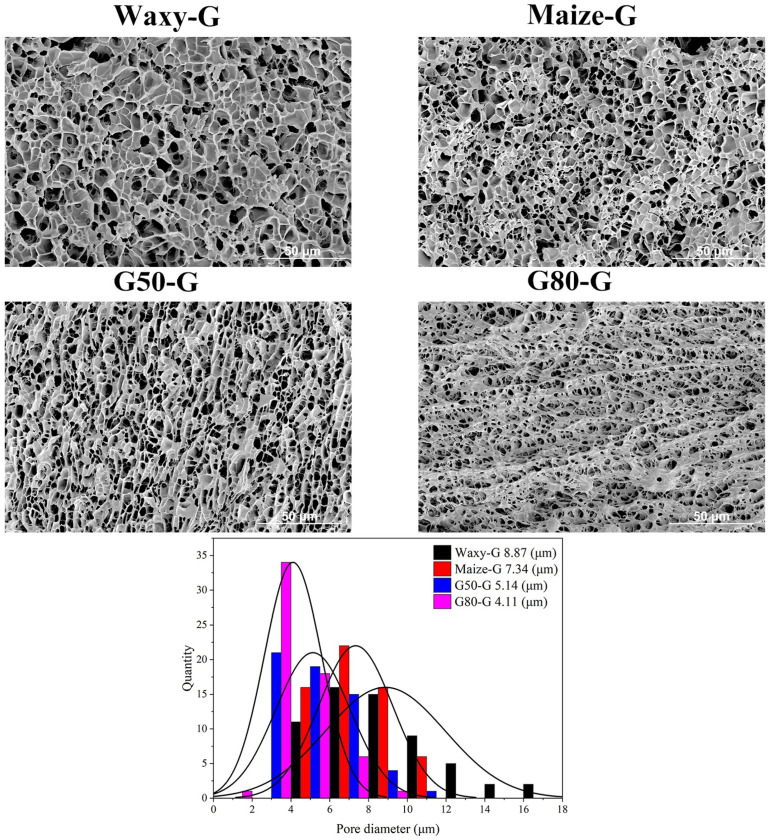
SEM images and pore size distributions of crosslinked hydrogels (Waxy-G, Maize-G, G50-G, and G80-G).

Figure 2 shows the cross-sectional morphologies and pore size distributions of the freeze-dried hydrogels. All the hydrogels exhibited a uniformly interconnected network structure. The pore diameters of all the hydrogels are listed in Table 2. Increasing the amylose/amylopectin ratio resulted in a smaller pore diameter. This was due to the higher amylose contents of G50 and G80, which offered more hydroxyl groups to form dynamic borate ester bonds with borate ions and intermolecular hydrogen bonds, leading to a greater degree of crosslinking and thus resulting in a strong network that could reduce the mobility of free water molecules and maintain the pore structure during ice crystal growth and sublimation [53].

### 3.2. Gel Fraction and Swelling Ratio

**Figure 3:**
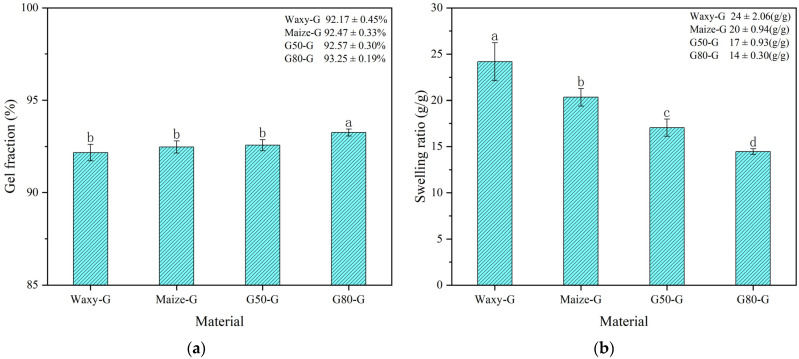
(**a**) Gel fractions of crosslinked hydrogels (Waxy-G, Maize-G, G50-G, and G80-G); (**b**) swelling ratios of crosslinked hydrogels (Waxy-G, Maize-G, G50-G, and G80-G). Values with different letters are significantly different (*p* < 0.05).

The effect of the amylose/amylopectin ratio on the gel fraction percentage is shown in Figure 3a. The gel fraction of the hydrogels increased from 92% to 93% with increasing amylose content. This was due to the steric hindrance effect of the highly branched amylopectin, which prevented the acrylamide from accessing the free radicals to be grafted to the starch chains and reduced the strength of the hydrogel [54]. Thus, the hydrogels originating from waxy had lower gel fractions. Figure 3b shows the relationship between the swelling ratio and the amylose/amylopectin ratio. The swelling ratio decreased from 24 g/g to 14 g/g with increasing amylose content. This phenomenon could be because the largely unbranched amylose component offered more hydroxyl groups to form borate ester bonds with borate ions and intermolecular hydrogen bonds, which led to a higher crosslink density of the hydrogels. This reduced the space between the polymer chains and restricted the expansion of the hydrogel network when in contact with water, resulting in a lower swelling ratio [55]. Therefore, the hydrogels prepared from low-amylose starches (waxy and maize) exhibited greater swelling ratios than the hydrogels prepared from high-amylose starches (G50 and G80).

### 3.3. Thermal Stability

**Figure 4:**
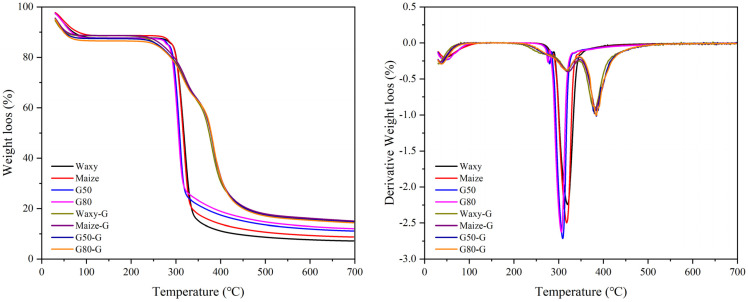
TGA and DTG of native starches and crosslinked hydrogels (Waxy-G, Maize-G, G50-G, and G80-G).

The TGA and derivative thermogravimetry (DTG) curves of the native starches and the Waxy-G, Maize-G, G50-G, and G80-G hydrogels are shown in Figure 4. All the native starches showed two decomposition stages, including the elimination of water molecules at 25–250 °C and the thermal decomposition of the main backbone of starch at 250–350 °C. The sequence of the peak decomposition temperature for the degradation of starch was waxy > maize > G50 > G80, indicating a greater thermostability of amylopectin-rich starches [56,57]. For the starch hydrogels, the TGA curves showed three-stage weight loss: weight loss in the first stage at 25–250 °C was due to water evaporation; weight loss in the second stage at 250–350 °C was attributed to the decomposition of the starch backbone; and the degradation of PAM grafted onto the starch chains in the third stage at 350–550 °C [47]. In addition, the weight residue of the borax-crosslinked hydrogels increased at the end of the heating compared to that of the native starches, and the decomposition temperatures of the hydrogels at stage two were higher than those of the corresponding starches, which was due to the strong bonding between the grafted polymer chains, borax, and starch chains, leading to a higher thermal stability of the hydrogels.

The percentage add-on (*AO*) and grafting ratio (*GR*) of the hydrogels are listed in Table 3. These grafting characteristics were calculated according to the percentage weight loss detected by the TGA [58]. The weight loss percentage of the starch hydrogels at stage 2 was recorded as *W_s_*_2_, while the weight loss percentage at stage 3 was recorded as *W_s_*_3_. The *AO* and *GR* were calculated from the following equations:

(3) $$AO(\%)=\frac{W_{s3}}{W_{s2}+W_{s3}}\times 100$$

(4) $$GR(\%)=\frac{W_{s3}}{W_{s2}}\times 100$$

The *AO* and *GR* increased from 66.03% and 194.34% to 68.06% and 213.06%, respectively, as the amylose/amylopectin ratio increased, which could be attributed to the highly branched amylopectin hindering the access of acrylamide to free radicals to be grafted to the starch chains and suppressing graft polymerization. On the other hand, the higher amylopectin contents of wax and maize reduced the mobility of the polymer chains and led to a high-viscosity system that could encapsulate free radicals in “cages” and hinder acrylamide from accessing free radicals, leading to a decrease in the amount of acrylamide involved in grafting reactions and reducing the AO and GR [59]. Therefore, the hydrogels prepared from low-amylose starches (waxy and maize) exhibited a lower AO and GR than the hydrogels prepared from high-amylose starches (G50 and G80).

### 3.4. Rheological Properties

**Figure 5:**
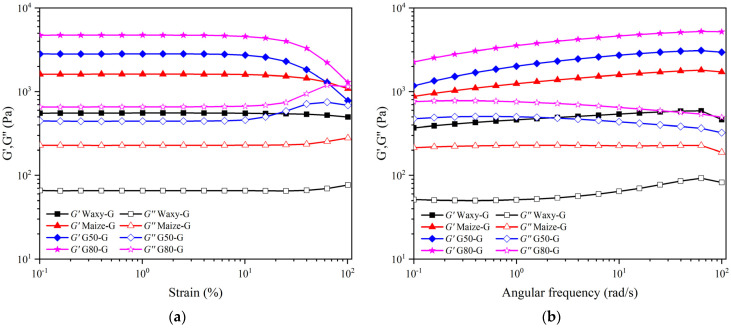
(**a**) Dynamic strain sweep curve at ω = 10 rad/s; (**b**) dynamic frequency sweep curve at γ = 1%.

The effect of the amylose/amylopectin ratio on the rheological properties of the starch hydrogels was studied. Figure 5a shows the curves for the storage modulus (*G*′) and loss modulus (*G*″) as a function of the strain amplitude (γ = 0.1–100%) at a frequency of 10 rad/s. Dynamic strain sweep tests were conducted to determine the linear viscoelastic region of the hydrogels, and the *G*′ values of all the hydrogels were relatively stable within γ = 1%, so γ = 1% was used for the dynamic frequency sweep tests to ensure that the deformation of the hydrogels was within the linear viscoelastic region [60]. The *G*′ value was always greater than the *G*″ value, indicating that all the hydrogels exhibited elastic-like behavior. The *G*′ and *G*″ pronouncedly increased with an increasing amylose/amylopectin ratio. The corresponding *G*′max values were 556.07, 1617.8, 2828, and 4748.7 Pa for the Waxy-G, Maize-G, G50-G, and G80-G hydrogels, respectively. An increase of almost 8.54 times in the *G*′max was generated for G80-G compared with Waxy-G. This was due to the largely unbranched amylose contents of G50 and G80, which provided more hydroxyl groups to form dynamic borate ester bonds with borate ions and intermolecular hydrogen bonds, leading to an enhanced crosslink density of the hydrogels. This leads to an increase in the *G*′ and *G*″.

The dynamic frequency sweep tests were recorded at angular frequencies (ω) ranging from 0.1 to 100 rad/s for each of the hydrogels at 1% strain, and the results are shown in Figure 5b. Over the entire ω range, the *G*′ values of all the hydrogels were greater than the *G*″ values, which suggested that the hydrogels were elastic. Unlike permanently crosslinked hydrogels, which demonstrated frequency-independent moduli, all the hydrogels exhibited frequency-dependent moduli, suggesting that these hydrogels were dynamically crosslinked [61]. In addition, the trends in the *G*′ and *G*″ for all the hydrogels were similar to those of the strain sweep tests. The corresponding *G*′max values were 589.76, 1804.1, 3100.6, and 5261.1 Pa for the Waxy-G, Maize-G, G50-G, and G80-G hydrogels, respectively, which could be explained by the fact that highly branched amylopectin reduced the mobility of the polymer chains and led to a high-viscosity system that restrained the acrylamide from accessing the free radicals on starch chains, resulting in a weak gel and thus leading to a decrease in the *G*′ and *G*″.

### 3.5. Self-Healing Property

**Figure 6:**
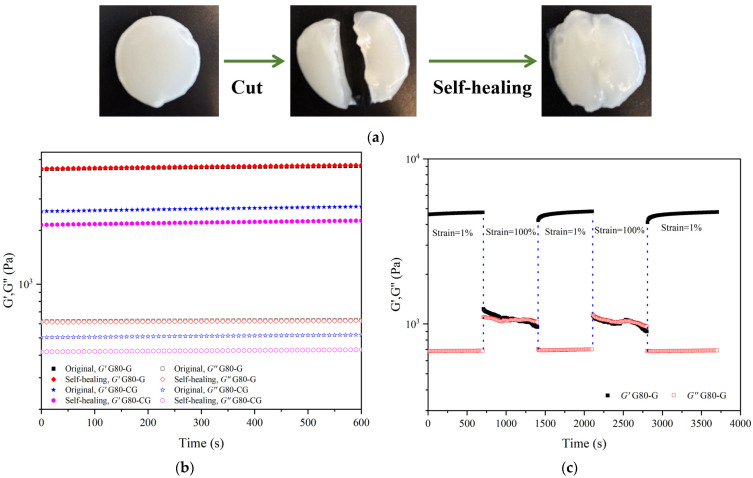
(**a**) Self-healing pictures of G80-G hydrogels; (**b**) *G*′ and *G*″ versus time for original and self-healing G80-G and G80-CG hydrogels after being cut; and (**c**) continuous step strain measurements of the G80-G hydrogel at strains of 1% and 100%.

The self-healing ability was also investigated by rheological tests, and G80-G was selected as a representative hydrogel, the results of which are shown in Figure 6. Figure 6a shows that the G80-G hydrogel was cut into two parts and immediately contacted in situ at room temperature to test the *G*′ and *G*″ of the hydrogel. The *G*′ (4434.8 Pa) and *G*″ (613.21 Pa) of the self-healing G80-G hydrogel were similar to the *G*′ (4407.1 Pa) and *G*″ (619.47 Pa) of the original G80-G hydrogel, indicating the full recovery of the inner structure of the hydrogel. Compared to the G80-G hydrogel, the G80-CG hydrogel could only recover part of its mechanical properties, which was due to the hydrogen bonds in the network. In Figure 6c, the hydrogel demonstrates its self-healing recovery under strain damage. At a low strain (γ = 1%), the hydrogel was solid, with a *G*′ of approximately 4611 Pa and a *G*″ of approximately 684 Pa. Subsequently, the *G*′ decreased from 4611 Pa to approximately 960 Pa under a high strain (γ = 100%), indicating that the mechanical stability of the hydrogel decreased. When the γ was decreased to 1%, the *G*′ and *G*″ returned to their original values immediately, indicating the self-recovery property of the hydrogel. All these results revealed the self-healing property of the hydrogel, which could be attributed to the hydrogen bonds and the dynamically reversible borate ester bonds.

### 3.6. Thermosensitivity Property

**Figure 7:**
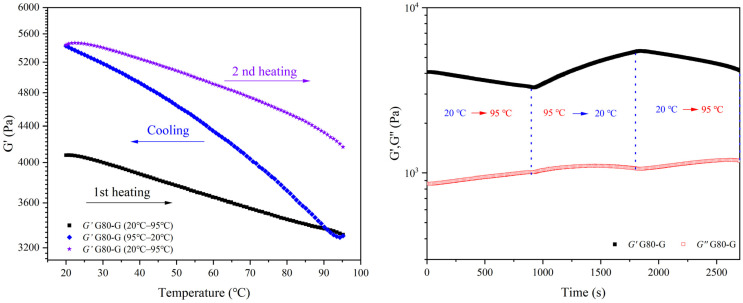
Temperature dependence of the *G*′ and *G*″ for the G80-G hydrogel during a heating–cooling–heating cycle at ω = 10 rad/s and γ = 1%.

The G80-G hydrogel was selected as an example to demonstrate its thermosensitivity, and the results are shown in Figure 7. During the heating–cooling–heating cycle, the *G*′ was always greater than the *G*″, suggesting the elastic-like behavior of the hydrogel. During the first heating process, the *G*′ decreased with the increasing temperature. In the following cooling process, the *G*′ increased rapidly from 95 to 20 °C, even higher than its original value, which might be caused by water evaporation inside the hydrogel. During the second heating process, when the temperature increased from 20 to 95 °C, the *G*′ decreased again. These results indicated the thermal responsiveness of the hydrogel, which could be explained by the reversible and exothermic interactions between the hydroxyl groups of the starch and borate ions [62].

## 4. Conclusions

In this work, corn starches with different amylose/amylopectin ratios were used as model materials to study the effect of amylose content on the grafting reactions and the performance of borax-crosslinked hydrogels. The hydrogels were simply prepared by grafting acrylamide onto starches, followed by crosslinking with borax. The results showed that the gel fraction and grafting ratio increased with an increasing amylose/amylopectin ratio, while the swelling ratio and pore diameter decreased with increasing amylose content. The increase in the *G*′ and *G*″ of the hydrogels verified the improved mechanical strength effect of a higher amylose content, and the *G*′ of the G80-G hydrogel was almost 8.54 times greater than that of the Waxy-G hydrogel. This was because unbranched amylose offered more hydroxyl groups to form dynamic borate ester bonds with borate ions and intermolecular hydrogen bonds, leading to an enhanced crosslink density of the hydrogels, while highly branched amylopectin hindered acrylamide from accessing the free radicals on starch chains, resulting in a weak hydrogel. Furthermore, benefiting from the dynamic borate ester bonds and hydrogen bonds, the hydrogels also demonstrated self-healing and thermally responsive properties. The G80-G hydrogel could recover to its original state after 100% shear strain. Overall, this work could help to understand the effect of starch microstructures on the properties of starch-based hydrogels and consequently broaden their practical applications in agriculture, sensors, and wastewater treatment.

## Figures and Tables

**Table 1:** Sample codes and composition.

Samples	Starch (g)	CAN (g)	Borax (g)	AM (g)
Waxy-G	5.00	0.70	0.50	15.00
Maize-G	5.00	0.70	0.50	15.00
G50-G	5.00	0.70	0.50	15.00
G80-G	5.00	0.70	0.50	15.00
G80-CG	5.00	0.70	-	15.00

**Table 2:** Pore diameter of crosslinked hydrogels (Waxy-G, Maize-G, G50-G, and G80-G).

Samples	Pore Diameter (μm)
Waxy-G	8.87 ± 3.09 ^a^
Maize-G	7.34 ± 1.91 ^b^
G50-G	5.14 ± 1.93 ^c^
G80-G	4.11 ± 1.48 ^d^

The values in the same column with different letters are significantly different (*p* < 0.05).

**Table 3:** “Percentage add-on” (AO) and grafting ratio (GR) of crosslinked hydrogels.

Samples	AO (%)	GR (%)
Waxy-G	66.03	194.34
Maize-G	66.25	196.27
G50-G	67.35	206.29
G80-G	68.06	213.06

## Data Availability

The data will be made available upon request.
